# Chemical, Bioactive, and Functional Characterization of a Protein Preparation from *Prunus padus* L. Flour

**DOI:** 10.3390/molecules30183766

**Published:** 2025-09-16

**Authors:** Izabela Kusak, Joanna Miedzianka, Agnieszka Nemś, Alicja Kosmenda, Szymon Wolny

**Affiliations:** 1 Department of Biotechnology and Food Microbiology, Wrocław University of Environmental and Life Sciences, 37 Chelmonskiego Street, 51-630 Wrocław, Poland; 122430@upwr.edu.pl; 2 Department of Food Storage and Technology, Wrocław University of Environmental and Life Sciences, 37 Chelmonskiego Street, 51-630 Wrocław, Poland; agnieszka.nems@upwr.edu.pl (A.N.); 121645@upwr.edu.pl (A.K.); szymon.wolny@upwr.edu.pl (S.W.)

**Keywords:** *Prunus padus* L. flour, protein preparation, antioxidant properties, amygdaline, functional properties

## Abstract

This study analyzed the chemical, functional, and bioactive properties of a protein preparation obtained from bird cherry (*Prunus padus* L.) flour. The extraction process significantly increased the protein content from 15.44 g/100 g to 39.72 g/100 g and altered the lipid composition, with an increase in saturated and polyunsaturated fatty acids. The protein preparation exhibited high solubility (76%) and water-binding capacity, demonstrating technological potential for use in the production of plant-based beverages and emulsions. Changes in color and emulsifying properties indicated its suitability for incorporation into colored food products. The analysis of total phenolic content, antioxidant activity, and amygdalin showed that the extraction process largely retained these bioactive properties, although individual phenolic compounds were not profiled. This study is the first to provide a comprehensive characterization of the protein fraction from *Prunus padus* L. flour, encompassing chemical, functional, and bioactive properties, thereby filling a significant gap in the literature. In summary, bird cherry flour is an attractive source of natural proteins and bioactive compounds, with potential applications in the food industry, while maintaining a favorable nutritional and functional profile. To date, no comprehensive chemical, functional, and bioactive characterization of the protein fraction from *Prunus padus* L. flour has been reported.

## 1. Introduction

In recent years, there has been a growing interest in plant-based protein ingredients, which are increasingly used in the food industry not only for their nutritional value and technological functionality but also for their potential health benefits [1]. In response to the need for alternative protein sources and functional ingredients, plants previously underused or neglected in traditional diets are gaining attention. The term ‘underutilized’ refers to plant resources that, despite their bioactive composition, have been historically neglected in food production. For *Prunus padus* L., this is largely due to its perception as an ornamental species rather than a food crop, the presence of antinutritional compounds such as cyanogenic glycosides, and the lack of well-established agronomic and processing practices. Compared to other economically representatives of the genus (e.g., almond and apricot), its potential in the food industry has therefore remained largely overlooked.

**Figure 1:**
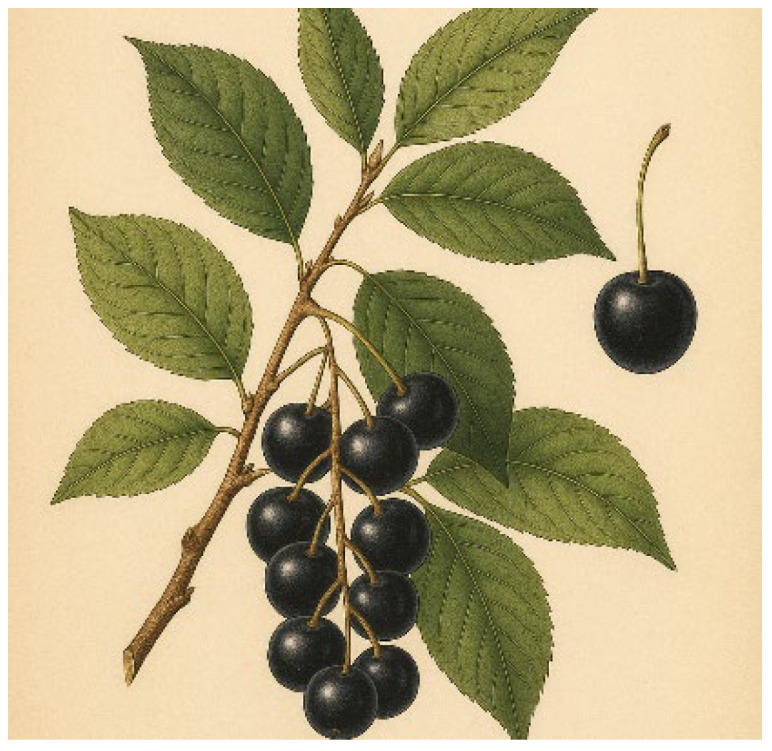
Plant species *Prunus padus* L. with its fruit (cherry). Own elaboration.

Previous studies on *Prunus padus* L. (Figure 1) have mainly focused on fruits, leaves, and bark, which are rich in polyphenols, flavonoids, and other phytochemicals exhibiting antioxidant, anti-inflammatory, antimicrobial, and antidiabetic activities [2,3,4]. In particular, fruit extracts have been shown to contain high levels of anthocyanins, phenolic acids, and flavonol glycosides, contributing to their antioxidant activity and vasoprotective effects [5,6]. Traditional uses of bird cherry fruits include infusions, syrups, and tinctures used for treating respiratory and circulatory ailments [6]. However, the protein fraction of the seeds remains poorly characterized, in terms of both its chemical composition and functional properties. This knowledge gap is especially important in light of the growing interest in novel plant-based protein ingredients with potential applications in functional foods and nutraceuticals.

It is worth noting, however, that some of these raw materials also contain antinutritional substances, such as cyanogenic glycosides. An example is amygdalin, which is found in the seeds of plants belonging to the genus *Prunus* and, upon enzymatic hydrolysis, can release toxic hydrogen cyanide (HCN). Despite their potential hazard, these compounds—in small doses—exhibit pharmacological properties, including antitussive and spasmolytic effects [7]. There is also growing interest in species previously treated as ornamental plants, whose fruits and seeds are rich in bioactive components. Such raw materials include the American bird cherry (*Prunus padus* L.), whose biological potential is still relatively poorly understood [5]. This plant, also known as bird cherry or ‘capulín’, has been used in traditional medicine to treat respiratory and circulatory ailments [6]. Bird cherry fruits have been used to prepare infusions, syrups, and tinctures.

Fruits and seeds of *P. padus* L. are rich in polyphenols, flavonoids, and anthocyanins, including compounds such as cyanidin-3-glucoside, chlorogenic acid, and quercetin derivatives. They also contain organic acids (e.g., malic and citric acids) and vitamins (including vitamin C and α-tocopherol), which contribute to their antioxidant, anti-inflammatory, and vasoprotective properties [5,6,8]. Previous studies have focused mainly on extracts from the fruits, leaves, and bark of this plant, while little is known about the protein fraction of the seeds—particularly regarding its composition functional properties.

An interesting derivative of *P. padus* L. is flour obtained from dried and ground fruit. It is distinguished by an intense almond–chocolate aroma, high fiber content, and natural antioxidants. Unlike almond or apricot kernel flour, it contains moderate amounts of protein and fat but is rich in polyphenols derived from the whole fruit—including the peel and the pit. Thanks to its specific processing method, it requires no refining, and its sensory qualities make it an attractive ingredient for use in functional foods [9]. In light of the above, the aim of this study was to chemically characterize and evaluate selected functional properties of a protein preparation obtained from bird cherry (*Prunus padus* L.) seeds. The results obtained may expand knowledge about the potential use of this plant in the food, pharmaceutical, and cosmetic industries.

## 2. Results and Discussion

### 2.1. Chemical Composition

The flour and protein preparation obtained from *Prunus padus* L. seeds exhibited significant differences in their chemical composition, reflecting the distinct nature of these materials and the processing methods applied (Table 1). The dry matter content was higher in the protein preparation (96.58 ± 0.19 g/100 g) than in the flour (91.26 ± 0.02 g/100 g), indicating a lower moisture content and a higher concentration of solids in the processed protein fraction.

The total protein content in the protein preparation (39.72 ± 0.21 g/100 g) was over 2.5 times higher than that in the flour (15.44 ± 0.21 g/100 g), confirming that the preparation may serve as a valuable ingredient in high-protein or functional food formulations. However, the protein concentration in the obtained preparation, although markedly higher than in the flour, remains relatively low. This may be explained by incomplete recovery of protein fractions due to the mild extraction conditions applied. Notably, the protein content of bird cherry seeds is considerably higher than that of the mesocarp of other stone fruits, such as plum (0.9%), apricot (*Prunus armeniaca* L., 1.4%), peach (*P. persica* L. Batsch, 0.9%), or grape (*Vitis vinifera*, 0.72%) [5]. In contrast, the average protein content in kernels is much higher, often exceeding 30%, and is comparable to that of apricot (37.4%), sweet cherry (*P. avium* L., 31.7%), sour cherry (*P. cerasus* L., 31.7%), nectarine (*P. persica* var. *nectarina*, 38.7%), peach (33.4%), and plum (*P. domestica* L., 35.9%) [10]. The protein content in the flour (15.44 g/100 g) suggests that the sample may have contained both kernel and pulp residues. Although the purity of the isolated protein was not further assessed by techniques such as SDS-PAGE, the protein content determined by the Kjeldahl method confirmed that the preparation was suitable for the intended analyses.

The fat content was moderately high in both materials, though slightly higher in the flour (11.76 g/100 g) than in the protein preparation (8.94 g/100 g), likely due to partial removal of lipophilic compounds during protein concentration. This reduction is consistent with the defatting effect of the aqueous extraction and precipitation process, which promote the separation of non-polar compounds into the supernatant or into sediment fractions rich in cell wall material.

The ash fraction, representing the oxides of mineral ions, was significantly higher in the protein preparation (8.53 %) than in the flour (4.78 %), indicating enrichment in mineral components. This increase likely results from the co-extraction of mineral salts bound to the protein matrix, as well as a concentration effect caused by the removal of carbohydrates and lipids during processing. Similar trends have been observed in protein isolates from other oilseed and kernel sources, where extraction processes resulted in a simultaneous enrichment in both protein and mineral matter due to the selective retention of inorganic compounds associated with proteins. Such a correlation between increased protein content and higher ash fraction in protein preparations has been previously reported in studies on plant-derived ingredients, highlighting the co-precipitation of mineral salts with protein fractions during typical extraction and precipitation methods [11].

Carbohydrates, calculated by difference (including sugars, starch, and other polysaccharides), were substantially higher in the flour (59.28 g/100 g) than in the protein preparation (39.39 g/100 g), suggesting the flour is more suitable as an energy source. Importantly, these values include all carbohydrate fractions such as starch, soluble polysaccharides, and sugars. Total sugars, determined analytically, constituted a sub-fraction of these carbohydrates, with markedly higher levels in the flour (16.88 g/100 g) compared to the protein preparation (1.09 g/100 g). In contrast, the protein preparation, characterized by its higher protein and mineral contents and lower carbohydrate concentration, may be more appropriate for the development of functional food products with a reduced glycaemic potential [12].

One of the most pronounced differences was observed in dietary fiber content. The protein preparation exhibited a remarkably high fibre content (36.32 g/100 g), over 3.5 times greater than that of the flour (9.9 g/100 g). This likely results from the co-extraction of cell wall polysaccharides during protein isolation. The fibre content of the flour is consistent with that of other seeds and nuts, such as hazelnuts (9–13%) or pumpkin seeds (12.1%) [13]. High dietary fiber intake is associated with improved glucose metabolism and reduced glycaemic response, making the protein preparation a promising candidate for fibre-enriched functional foods, particularly for individuals with impaired carbohydrate metabolism [14,15].

Regarding sugars, *P. padus* L. seeds contain glucose and fructose as the predominant sugars, with sucrose present in minor amounts. The analysis revealed a significant difference in sugar content between the two materials. The flour contained 16.88 g/100 g of total sugars, constituting a substantial fraction of its carbohydrate content, whereas the protein preparation had only 1.09 g/100 g. Reducing sugars followed a similar pattern, with 12.69 g/100 g in the flour and 0.82 g/100 g in the preparation. These differences were statistically significant (*p* < 0.05). Reducing sugars play a key role in non-enzymatic browning reactions (the Maillard reaction) and strongly influence the glycaemic index of food products. The relatively high sugar content in the flour is consistent with the typical composition of cereal- and seed-derived flours [16].

### 2.2. Selected Bioactive Compounds and Antioxidant Capacity

Table 2 presents the total phenolic content (TPC), antioxidant activity (ABTS assay), and amygdalin content in the flour and the protein preparation obtained from American bird cherry (*Prunus padus* L.). Polyphenols, as natural antioxidants, play an important role in the prevention of lifestyle diseases by neutralizing free radicals and protecting against oxidative stress. They also support the proper functioning of the cardiovascular system by improving the elasticity of blood vessels, lowering blood pressure, and reducing the oxidation of LDL cholesterol, which is crucial for the prevention of cardiovascular disease [3,17].

TPC did not differ statistically significantly (*p* > 0.05) between the flour (15.38 mg GAE/g DM) and the protein preparation (15.31 mg GAE/g DM). This range corresponds well with the results obtained for *Prunus spinosa* L., in which TPC values were 4.70–37.23 mg GAE/g DM in ultrasonic extraction [18] and 12.17–20.94 mg GAE/g fresh fruit in classical extracts (Phenolic composition, antioxidant, and antimicrobial activity of the extracts from *Prunus spinosa* L. fruit). Maintaining a comparable level of phenolic compounds after the protein isolation process suggests that the procedure used did not lead to their significant degradation or loss.

Antioxidant activity determined by the ABTS method also showed no significant differences between samples (*p* > 0.05). Numerically, the protein preparation reached a slightly higher value (29.26 µmol Trolox/g DM) compared to the flour (26.36 µmol Trolox/g DM). However, this difference was not statistically significant and should be verified using complementary antioxidant assays (e.g., DPPH, FRAP, ORAC), which would provide a more comprehensive assessment of the antioxidant potential. In studies on cherry fruit, antioxidant activity is observed in the range of 73.2 to 89.1 µg Trolox per g of fresh fruit, with higher values recorded in darker colored fruits. This means that varieties with higher polyphenol content also exhibit stronger antioxidant activity, which translates into their potential applications in the production of functional food products and nutraceuticals [19].

Polyphenols present in the raw material can form complexes with proteins, which affect their stability, bioavailability, and antioxidant activity. These interactions can occur both reversibly (e.g., through hydrogen bonds) and irreversibly, leading to the formation of stable covalent complexes. This is important in the context of functional foods, as such complexes can demonstrate increased resistance to oxidative degradation and prolonged antioxidant activity. Furthermore, selected amino acids present in proteins, such as tyrosine, cysteine, and tryptophan, can enhance this effect through synergistic free radical-scavenging mechanisms. The antioxidant activity observed in the protein preparation may therefore result from the presence of both natural phenolic compounds and antioxidant peptides, which may have been formed during protein extraction or isolated with the protein fraction [20], as well as possible interactions between these components [21,22]. These peptides demonstrate the ability to chelate transition metals° and neutralize free radicals, making them desirable functional ingredients in food. Protein-polyphenol complexes not only influence the structure of proteins but also alter their functional properties.

The greatest differences between the analyzed samples were noted in the content of amygdalin—a cyanogenic glycoside characteristic of the seeds of plants belonging to the genus *Prunus* (Table 2). In bird cherry flour, its content was 1.72 mg/g, whereas in the protein preparation obtained from it, it was over 10-fold lower (0.15 mg/g), which corresponds to a reduction of approximately 90%. This significant reduction in the concentration of this compound can be explained by its moderate solubility and instability under alkaline conditions, as well as partial degradation due to the effects of temperature and enzymatic action during protein extraction. According to the study by Bolarinwa et al. [23], even short-term storage of apple juice at room temperature before freezing causes a decrease in the amygdalin content by 11–19%, confirming the susceptibility of this glycoside to degradation. Additionally, plant enzymes, such as β-glycosidase, can catalyze the degradation of amygdalin, and milling the raw material promotes the hydrolysis of this compound by facilitating the contact of glycosides with enzymes [24]. From a toxicological point of view, reducing the amygdalin content in the protein preparation is beneficial, as its excess can lead to the release of hydrogen cyanide in the gastrointestinal tract as a result of enzymatic hydrolysis [25]. For comparison, the amygdalin content in bird cherry flour is lower than in cherry seeds (2.68 mg/g), apricot (14.37 mg/g), and plum (17.49 mg/g) [26]. The toxic dose of cyanide for humans ranges from 0.5 to 3.5 mg/kg of body weight, and 1 mg of amygdalin releases an average of 0.24 mg of cyanide. This means that 875 mg of amygdalin can be equivalent to 210 mg of cyanide—a potentially toxic dose for a 70 kg person. Considering that 1 mg of amygdalin releases approximately 0.24 mg of HCN, and that the protein preparation contains 0.15 mg of amygdalin per gram, the maximum amount of product that would not exceed the toxic dose for a 70 kg person ranges approximately from 972 g to 6806 g, depending on the assumed toxic threshold (0.5–3.5 mg HCN/kg body weight). In practice, it is recommended to limit amygdalin consumption to a minimum to reduce the risk of toxic effects.

### 2.3. Amino Acid Composition

The amino acid profile of bird cherry (*Prunus padus* L.) flour and the protein preparation obtained from it was compared based on the data presented in Table 3. The obtained results indicate significant quantitative differences in the content of individual amino acids between the tested samples, demonstrating the effectiveness of the protein isolation process and its impact on nutrient concentrations. The observed differences in amino acid content between the flour and the protein preparation can be attributed to the concentration effect during protein isolation. While the protein preparation is derived from the flour, the extraction process removes a significant portion of non-protein components, such as carbohydrates and lipids, thereby enriching the protein fraction. Consequently, the total amino acid content per gram is higher in the protein preparation than in the original flour. Partial degradation of certain amino acids, such as methionine and cysteine, may also occur due to processing conditions, further influencing the profile. These changes reflect not the formation of new amino acids, but the selective enrichment and concentration of the existing protein fraction.

The total amino acid content in the protein preparation was 345.40 mg/g, almost three times higher than in the flour (122.05 mg/g), confirming the effective enrichment of the protein fraction. This applies to both exogenous amino acids (IAA) and endogenous amino acids (DAA). Among the exogenous amino acids, the protein preparation contained the most leucine, isoleucine, phenylalanine, threonine, and valine. These contents are important from a nutritional perspective, as branched-chain amino acids (BCAAs) play key roles in the synthesis of body proteins and the regulation of muscle metabolism. A similar pattern of amino acid content was described by García-Aguilar et al. [10], who showed that glutamic acid and leucine were the dominant amino acids in raw and roasted bird cherry seeds. These data also align with analyses conducted by Mustafa et al. [13], who examined the amino acid profile of flours from detoxified apricot, peach, and mango seeds. Their results also indicated a predominance of leucine, valine, and phenylalanine among the exogenous amino acids, and glutamic acid and aspartic acid among the endogenous amino acids. Low methionine and cysteine content were also characteristic of these raw materials, which is consistent with the results of this study, where their levels in the protein preparation were lower than in the flour (2.57 vs. 2.88 mg/g and 0.18 vs. 0.33 mg/g, respectively). The low stability of these compounds under processing conditions may be responsible for their partial degradation during isolation.

Among the essential amino acids, the high content of glutamic acid (74.41 mg/g) was particularly noteworthy. This acid is responsible for sensory properties (umami taste) and influences the functional properties of proteins. Similar values were found in the analysis of peaches conducted by Sun et al. [27], where glutamic and aspartic acids also dominated the fruit’s amino acid composition. The high content of glycine, alanine, and proline in the protein preparation further enhances its potential as a functional food ingredient.

Importantly, the high levels of glutamic acid and leucine increase the potential of this protein preparation for application in high-protein foods targeted at athletes or older adults, as these amino acids support muscle protein synthesis, recovery, and maintenance of lean body mass. Bird cherry proteins also demonstrate very good in vitro digestibility, estimated at approximately 88% [10], making them comparable to proteins from almonds or pumpkin. This bioavailability, combined with a favorable amino acid profile, may indicate the preparation’s high potential as an alternative protein source in food products with high nutritional value.

### 2.4. Fatty Acid Composition

In the fatty acid profile (Table 4) of both flour and protein preparation from American bird cherry (*Prunus padus* L.), the dominant compound was erucic acid (C22:1, cis-13-docosenoic acid), whose content was 74.55% and 68.16% in the samples, respectively. Such a high concentration of C22:1 is rarely found in plant materials and occurs mainly in some varieties of plants from the Brassicaceae family, such as mustard (*Brassica juncea*), industrial rapeseed (*Brassica napus*), Abyssinian cabbage (*Brassica carinata*), or broccoli (*Brassica oleracea*) [28,29]. The C22:1 content in mustard seeds and industrial oil can reach as much as 45–50% [30], and in *B. carinata* it can exceed 40% [31]. In most plant materials used for food purposes, the erucic acid content is much lower, typically <2%. The high C22:1 content in the analyzed samples is a distinctive feature, but from a toxicological point of view, it may raise concerns. The European Food Safety Authority (EFSA) has established an acceptable daily intake (TDI) of erucic acid at 7 mg/kg body weight [28], due to the possibility of accumulation in heart muscle tissue and potential cardiotoxicity at high doses. However, some reports also indicate a beneficial effect of C22:1 on lipid metabolism and inflammation under controlled conditions [32].

In addition to erucic acid, significant amounts of eicosenoic acid (C20:1) were detected in the flour and protein preparation—15.51% and 16.61%, respectively—and oleic acid (C18:1)—5.35% and 6.65%, respectively. The total share of monounsaturated fatty acids (MUFA) in the lipid profile of both samples was very high, reaching 96.94% for the flour and 92.92% for the protein preparation. Such a large predominance of MUFA, including long-chain monounsaturated fatty acids, is a phenomenon rarely observed in plant raw materials, and even more so in protein isolates. A similar MUFA profile was demonstrated only by oils from selected mustard varieties and broccoli seed oil [30,31].

As a result of the protein extraction process, the content of saturated fatty acids (SFA) increased from 2.01% in the flour to 5.34% in the preparation. Palmitic acid (C16:0) and stearic acid (C18:0) accounted for the largest share. Simultaneously, the content of polyunsaturated fatty acids (PUFA) increased from 1.98% to 4.16%, mainly due to the increased content of α-linolenic acid (C18:3n3) and linoleic acid (C18:2). The increased PUFA and SFA content in the preparation may result from differences in lipid retention during the extraction and defatting stages. According to previous studies, these differences may affect the selective retention of protein-bound lipid fractions [33].

The obtained fatty acid profile—despite the dominance of erucic acid—may have functional and technological significance, particularly in the context of the preparations’ stability (due to the presence of long-chain MUFAs) and their potential health-promoting effects (contribution of n-3 PUFAs). However, further research is necessary on the bioavailability and safety of such high doses of C22:1 (with long-term consumption). Therefore, the presence of erucic acid in *P. padus* seed-derived preparations should be considered both a limitation and a potential advantage. From a toxicological perspective, its high content requires careful evaluation of intake levels and may restrict direct application in commonly consumed foods. On the other hand, the distinctive lipid profile, dominated by long-chain MUFAs, could provide technological benefits, such as improved oxidative stability and specific functional properties valuable in selected formulations. Controlled use in specialized food products or nutraceuticals, with attention to safety guidelines, may thus represent a rational direction for further research and potential applications.

### 2.5. Functional Properties

#### 2.5.1. Solubility, Water- and Oil-Absorption Capacity

Functional properties, such as solubility and water and oil binding capacity, play a key role in food technology, influencing the texture, stability, and sensory quality of food products. This study compared these selected parameters for bird cherry (*Prunus padus* L.) flour and the protein preparation obtained from it (Table 5).

The Protein Solubility Index (PSI) is a key functional parameter influencing the technological applications of plant proteins in aqueous and colloidal products. In the presented study, a protein preparation from bird cherry seeds achieved significantly higher water solubility (~76%) than raw flour (~28%). The literature confirms that protein isolates from oil plants often exhibit PSI above 60–70%, while their raw flours are often below 30%. For example, in the case of hemp seed protein isolate (HPI), solubility was low in a neutral environment (≤20%), but increased to approximately 64–69% at pH 11–12, which was associated with the release of surface charge and reduced protein aggregation (Hemp Protein Isolate, PSI = 64–69% at pH 11–12) [34]. Similarly, in the case of a flaxseed preparation, studies indicate that protein isolate obtained by alkaline extraction and acid precipitation achieves solubility values exceeding 50% at neutral and slightly alkaline pH, while raw cake (flour) exhibits solubility of 20–30% [35]. Our PSI values are consistent with literature ranges for plant materials with high protein purity in the isolate form and low purity in the raw flour form. A high PSI for the preparation increases its functional potential, particularly in formulations such as emulsions, beverages, and highly aqueous systems, where full protein solubility is essential to ensure the proper structure and texture of the product. Furthermore, Tang et al. [36] emphasize that the high solubility of isolated proteins is closely related to their ability to form stable emulsions and foams, which is consistent with observations regarding the bird cherry protein preparation. The high PSI value obtained for the protein preparation suggests the good quality of the protein fraction and its potential technological usefulness, especially in aqueous or colloidal products such as plant drinks, emulsions, or special-purpose foods.

Water-absorption capacity (WAC) expressed per weight of the preparation was significantly higher for the flour (2.72 g/g) than for the protein fraction (2.15 g/g) (Table 5). An inverse relationship was observed after conversion to protein content: the protein preparation showed a higher WAC (0.85 g/g protein) than the raw flour (0.42 g/g protein). This suggests that despite a lower total water-binding capacity per weight of the product, the protein fraction has a higher affinity for water per protein unit. This may be due to structural modifications of the proteins (e.g., partial unfolding or exposure of hydrophilic residues) that occur during the extraction process [37,38].

In turn, the oil-absorption capacity (OAC) was significantly higher in flour (5.42 mL/g) than in the protein preparation (1.67 mL/g), both per product mass and per protein unit (0.84 mL/g and 0.66 mL/g, respectively). This may be related to the presence of carbohydrates, fiber, and residual lipids in flour, which support fat absorption due to their porous structure and the presence of non-polar groups [39]. Protein preparations, on the other hand, are characterized by a more hydrophilic character and a lower lipid-binding capacity [40]. Similar OAC values for flour were also observed for raw materials with a similar lipid composition and the presence of non-starch fractions, such as chia seed flour (ca. 5.4 mL/g) [41] or hemp seed flour [36]. However, the WAC of protein preparations obtained from legumes (e.g., pea protein, soy protein) shows comparable or slightly lower values (0.6–0.9 g/g protein), depending on the extraction and modification method [42]. Water absorption capacity (WAC) is expressed as grams of water per gram of sample (g/g), while oil absorption capacity (OAC) is expressed as milliliters of oil per gram of sample (ml/g), which is consistent with common practice in the literature [39,40].

#### 2.5.2. Emulsifying Properties

The emulsifying properties of the flour and the bird cherry protein preparation showed significant differences in emulsifying activity and emulsion stability (Table 6). The flour had a higher emulsifying activity (EA) (82.22%), while the protein preparation achieved a lower value (47.53%). This may be due to the removal of amphiphilic components, such as starch and fiber, during the protein isolation process. The high EA value of the flour indicates its good ability to stabilize and bridge lipophilic and hydrophilic phases.

Emulsifying stability (ES) was significantly higher for the protein formulation (63.22%) than for the flour (35.56%). This result suggests that isolated proteins stabilize emulsions more effectively, which can be attributed to their higher purity, higher concentration, and their ability to form ordered interfacial films that prevent fat droplet coalescence. The emulsion stability of the formulation is also supported by its hydrophobic-hydrophilic profile, moderate OAC values, high WAC (per gram of protein), and the presence of unsaturated fatty acids. In particular, the presence of monounsaturated fatty acids (MUFA), such as erucic acid (C22:1, 68.16%), eicosenoic acid (C20:1, 16.61%), and oleic acid (C18:1, 6.65%), favors the formation of stable systems that are resistant to coalescence and destabilization. According to the results of Zheng et al. [43], unsaturated fatty acids improve the stability of the protein interface by increasing the surface charge and repulsive forces between fat droplets. Furthermore, the presence of saturated fatty acids (SFA) in the preparation (5.34%) may support the formation of stiffer and more durable interfacial films. In the literature, EA values for protein isolates from oil plants, such as mustard, hemp, or flax, are usually in the range of 40–60%, while ES values are in the range of 55–75%, confirming that the properties of the bird cherry protein preparation are close to typical ranges [44].

The stability of the emulsion obtained from flour (35.56%) indicates its susceptibility to flocculation and coalescence. Studies have shown that different plant proteins can affect emulsion stability to varying degrees, depending, among other things, on their interactions with polyphenols [45]. Similar EA and ES values were reported in the work of Vázquez-Ovando et al. [46] for proteins from Spanish sage (*Salvia hispanica* L.), where EA was 50–56% and ES reached 92% in a slightly alkaline environment. The authors attributed this effect to the ability of proteins to form ordered layers at the phase interface.

Protein interactions with polyphenols may additionally influence emulsification properties. Polyphenols, thanks to their antioxidant properties, can strengthen protein structure and improve both EA and ES. Although precise data on the polyphenol content of bird cherry are still limited, their potential presence could have affected the obtained results. Noncovalent interactions of these compounds with proteins—though not always beneficial for stability—can, under appropriate conditions, lead to beneficial functional changes. Anthocyanins may be of particular importance, improving the antioxidant and emulsifying properties of protein preparations.

#### 2.5.3. Colour

**Figure 2:**
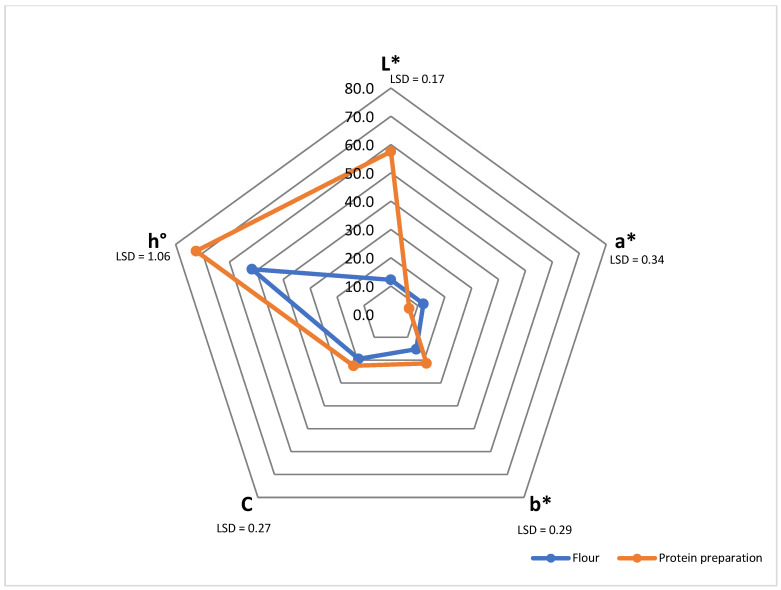
Colour of bird cherry flour and protein preparation. L*—lightness; a*—redness to greenness; b*—yellowness to blueness; C*—chroma colour; h—hue angel.

Color analysis of the samples (Figure 2) revealed significant differences between the flour and the bird cherry protein preparation in all analyzed parameters. The protein preparation was characterized by a significantly higher lightness value L* (57.56) compared to the flour (12.22), indicating a significant lightening of the material after the protein isolation process. This increase in lightness is typical of protein extraction and purification processes, which result in the removal of colored components such as phenols, natural pigments (e.g., anthocyanins), or dietary fiber particles, all of which are responsible for the dark color of the raw material [47]. The value of the a* parameter responsible for the red color intensity was significantly higher in the flour (12.08) than in the protein preparation (6.80), indicating a reduction in reddish components—which may originate from the Maillard reaction or be present in the form of natural pigments. At the same time, the value of the yellow component b* was higher in the preparation (21.42) than in the flour (15.24), possible due to the release or concentration of yellow compounds, such as some carotenoids or lipid residues.

The observed differences in chroma (C*) and hue angle (h) further confirm the change in the sample’s color characteristics. The protein preparation exhibited higher chroma (C* = 22.47) and a larger hue angle (h = 72.39°) than the flour (C* = 19.45; h = 51.62°), indicating a shift in color perception toward a more intense, light yellow hue. The increased hue angle also suggests that the color of the protein preparation is closer to yellow than red, which is beneficial for applications in light or neutral-colored food products. Such changes in color parameters resulting from protein extraction are consistent with observations for other plant materials, e.g., in isolates from pea, soybean, or flax, where the fractionation process also led to significant lightening of the material and modification of the color profile [48].

The process of extracting protein from bird cherry seed flour significantly affects color parameters, resulting in a lighter product, less red and more yellow. Compared to other plant-based protein preparations, bird cherry protein has comparable or slightly lower brightness but higher color saturation. This can be of technological importance, for example, in the production of light-colored food products where minimal coloring is required.

## 3. Materials and Methods

### 3.1. Materials and Chemicals

The research material was bird cherry flour (*Prunus padus* L.), which was purchased from a retailer (Siberian Green EU, Tallinn, Estonia). The producer does not provide information on the exact location and time of fruit harvesting, which should be considered a potential factor influencing the product’s chemical composition. Bird cherry flour was obtained by drying ripe fruit and then finely grinding it, usually with the seeds still attached, to preserve its full nutritional value and characteristic aroma. The raw material was sieved to standardize its granulation.

Amygdalin standard, ethanol, and HPLC-grade acetonitrile were purchased from Sigma-Aldrich (Darmstadt, Germany). Water was prepared using Millipore Milli-Q purification system (Merck KGaA, Darmstadt, Germany). All other chemicals used in the experiment were of analytical grade.

### 3.2. Preparation of Protein Preparation

Proteins from bird cherry flour were isolated by alkaline extraction. A 0.5 M NaOH solution was used in a 1:15 ratio at room temperature for 23 h, and the samples were continuously mixed with a magnetic stirrer. The extraction time of 23 h was selected based on preliminary experiments, in which protein yield was monitored every 2 h over a 24 h period; no significant differences were observed between 23 and 24 h, indicating that 23 h was sufficient for maximum protein recovery. After this time, the samples were centrifuged at ≈1314× *g* using an MPW-351 centrifuge (Heraeus Sepatech, Osteorode, Germany) for 30 min to separate the extract containing dissolved protein from the precipitate remaining after protein extraction from bird cherry seeds. The extracted protein was coagulated at its isoelectric point (pI = 4.5) using 0.1 M HCl at room temperature for 15 min. This value is commonly applied in protein isolation protocols since many plant seed proteins exhibit minimal solubility within the pH range of 4–5. The resulting protein precipitate was centrifuged at ≈1957× *g* for 30 min, yielding coagulated protein and a coagulated supernatant. The centrifugation speeds were selected based on previous studies on plant protein extraction [49]. The wet protein pellet was dried by lyophilization (Christ Alpha 1–4 LSCplus lyophilizer (Osterode am Harz, Germany) to obtain a dry protein preparation. The dried protein preparations were sieved to homogenize the structure. The prepared preparations were stored in zip-lock bags at −18 ˚C until further analysis. Alkaline extraction was selected as a commonly used and efficient method for seed proteins. However, it should be noted that the extraction method may affect protein yield and functional properties, and alternative approaches such as enzymatic, salt-based, or assisted extraction methods could be considered in future studies.

### 3.3. Chemical Composition

Proximate chemical analysis of the flour and protein preparation was performed according to AOAC methods [50]. The dry matter (DM) content was determined based on the weight loss observed during thermal drying at 105 °C until a constant weight was achieved. Total nitrogen was determined by the Kjeldahl method using a Büchi Distillation Unit K-355 (Büchi Labortechnik AG, Flawil, Switzerland). A nitrogen-to-protein conversion factor of 6.25 was used to calculate total protein, as standard. Fat content was determined by the Soxhlet extraction method using diethyl ether after hydrolysis of the sample with 4 mol L^−1^ HCl, in a Büchi B-811 (Büchi Labortechnik AG, Flawil, Switzerland) apparatus. The ash content was determined by adding 1 g of the sample to a crucible, incinerating it in a muffle furnace at 550 °C, and weighing the residue. Total carbohydrate content (on a dry weight basis) was calculated by difference [100 − (protein + lipids + ash + crude fiber)]. Crude fibre content was determined according to the Hennenberg and Stohmann method by acid hydrolysis with H_2_SO_4_ followed by alkaline hydrolysis with NaOH. Total and reducing sugars were determined by a colorimetric method with 3,5-dinitrosalicylic acid (DNS) [51]. Data are reported as the mean value ± standard deviation (SD) for three measurements.

### 3.4. Total Polyphenolic Compounds and Antioxidant Activity

Extraction procedure: Approximately 1 g of the material was mixed with 10 mL of 80% methanol in deionized water containing 1% HCl. The samples were then sonicated twice for 20 min at 20 °C (800 W, 40 Hz, Sonic 6D, Polsonic, Warsaw, Poland) and subsequently stored for 24 h at 4 °C. Following this treatment, the extract was centrifuged for 10 min at 19,000× *g* and used for further analyses.

Total polyphenolic compounds: Total polyphenols were quantified using the Folin–Ciocalteu method [52]. In brief, 100 µL of the extract was combined with 2000 µL of distilled water, 200 µL of Folin–Ciocalteu phenol reagent, and 1000 µL of 20% sodium carbonate solution. The mixture was incubated for 1 h in the dark at 20 °C. Absorbance was measured at 765 nm using a UV-2401 PC spectrophotometer (Shimadzu Corp., Kyoto, Japan). Total polyphenols were calculated from the calibration curve (y = 23.35 × Abs − 1.675) and expressed as milligrams of gallic acid equivalents (GAE) per gram of dry matter (mg GAE/g DM).

Antioxidant activity: The radical-scavenging activity was assessed using the ABTS assay, according to Ref. [53]. Briefly, 30 µL of the extract was mixed with 3 mL of ABTS reagent. After 6 min of reaction, absorbance was measured at 734 nm (UV-2401 PC spectrophotometer, Shimadzu, Kyoto, Japan). The ABTS activity was calculated using Trolox (TE) as a reference, and results were expressed as micromoles of Trolox equivalents per gram of sample (µmol TE/g DM).

### 3.5. Amygdalin Content

Sample Preparation: 1.5 g of each sample was weighed into a round-bottom flask (500 mL). 50 mL of ethanol was added, and the mixture was boiled under reflux for 100 min. The extract was filtered through Whatman No.1 filter paper and evaporated using a rotatory evaporator. Dry residue was dissolved in 5 mL of water and 1 mL of solution was filtered through syringe filter (Pureland, hydrophobic PTFE, 0.45 μm) (Chemland, Stargard, Poland). Amygdalin content in samples were determined by RP-HPLC, using Shimadzu Prominence—and LC-2030C Plus apparatus equipped with LC-2030 UV detector with wavelength set at 214 nm (Shimadzu Corporation, Kyoto, Japan). Supelcosil LC-18 (250 mm × 4.6 mm, 5 μm) column was placed in column oven set to 40 °C. The mobile phase consisted of acetonitrile and water (20:80 *v*/*v*) and peak separation was achieved with isocratic elution at the flow rate of 1 mL/min. Injection volume was 5 μL [23].

### 3.6. Amino Acid Composition

The analysed samples were acid-hydrolyzed [54] and analyzed using an AAA400 automatic amino acid analyser (INGOS s.r.o., Prague, Czech Republic). A two-wavelength photometer (440 and 570 nm) served as the detector. The metallic column, packed with the ion exchanger Ostion LG ANB (INGOS s.r.o., Prague, Czech Republic), measured 250 × 4.0 mm, with the column temperature maintained at 40–70 °C, and the detector temperature set at 121 °C. Amino acids were quantified using the ninhydrin method. Glutamine and asparagine were expressed as glutamic acid and aspartic acid, respectively. Tryptophan was not analysed. Calculations were performed using the computer program Chromulan (Pikron s.r.o., Prague, Czech Republic). All amino acid profiles were analysed in duplicate.

### 3.7. Fatty Acid Profile

Fatty acid methyl esters (FAMEs) were prepared following the procedure described by the AOAC (Association of Analytical Communities, AOAC Official Method, Gaithersburg, 1996) [50]. Aliquots of 0.1 mL of lipid extract from each sample were esterified with 2 mL of methanolic 0.4 M NaOH solution by refluxing for 10 min at 80 °C. After the addition of 4 mL of BF3-etherate, the samples were boiled for 5 min. The FAMEs were extracted with hexane (2.0 mL). For drying, 1 mL of NaCl was added and allowed to settle to clarify the hexane layer, after which the upper phase was transferred to a specific vial

Qualitative and quantitative analysis of fatty acid composition was performed by gas chromatography using an Agilent 7820A gas chromatograph (Agilent, Santa Clara, CA, USA). Fatty acid methyl esters were prepared with BF3 in methanol as the methylating agent. A ZB-WAX capillary column (30 m, internal diameter 0.25 mm, film thickness 0.25 µm) was used. The detector (FID) temperature was set at 280 °C, and the injection temperature was set at 260 °C with a split ratio of 1:100. The column temperature was initially 100 °C and was increased to 180 °C at 2 °C/min, then further heated to 240 °C at 6 °C/min and held for 10 min. Helium was used as the carrier gas. Peaks were identified based on their retention times using authentic standard fatty acid methyl esters, and all samples were run in duplicate.

### 3.8. Functional Properties

#### 3.8.1. Protein Solubility Index

The protein solutions were gently shaken at room temperature for 30 min and subsequently centrifuged at 4000× *g* for 15 min (Rotofix 32A, Hettich, Tuttlingen, Germany). The protein content of the supernatants was determined using the Kjeldahl method. Protein solubility was calculated as follows:

PSI = (PCS/TPC) × 100 (%)(1)

where PCS is the protein content in the supernatant after centrifugation, and TPC is the total protein content in the sample. PSI at different pH levels was determined in three analytical replicates per sample.

#### 3.8.2. Water- and Oil-Absorption Capacity

The water-absorption capacity (WAC) of bird cherry flour and protein preparation was determined using a published method [49,55]. Specifically, 0.5 g of each sample was mixed with 5 mL of distilled water, shaken for 15 min at room temperature, and centrifuged at 4000× *g* for 15 min (Rotofix 32A, Hettich, Tuttlingen, Germany). The supernatant was discarded, and the residue was oven-dried at 50 °C for 30 min. WAC was calculated as grams of water absorbed per gram of sample and per gram of protein. Each measurement was performed in triplicate.

The oil-absorption capacity (OAC) of bird cherry seed flour and protein preparation was determined using the method described in [49], with slight modifications. For each test, 0.5 g of each sample was weighed and mixed with 15 mil of rapeseed oil using a multi-station shaker for 30 min at room temperature. After centrifugation at 4000× *g* (Rotofix 32A, Hettich, Tuttlingen, Germany) for 10 min, excess oil was removed, and OAC was expressed as milliliters of oil absorbed per gram of sample. Each measurement was performed in triplicate.

#### 3.8.3. Emulsifying Properties

Emulsification activity (EA) and stability (ES) were estimated by the method described by Miedzianka et al. [54]. Protein suspensions were prepared by dissolving 0.5 g of each sample in 10 mL of distilled water. Then, 10 mL of rapeseed oil was added and mixed using a T25 basic ULTRA-TURRAX^®^ homogenizer (IKA-Werke GmbH & Co. KG, Staufen im Breisgau, Germany) for 1 min at 20.000 rpm. Then, they were centrifuged at 3000× *g* for 10 min. Emulsion stability was determined by centrifugation after heating at a temperature of 80 °C for 30 min.

EA = (a/b) × 100 (%)(2)

where a is the height of the emulsified layer in the tube and b is the height of the total contents in the tube.

ES = (c/d) × 100 (%)(3)

where c is the height of the emulsified layer after heating and d is the height of the total contents after heating.

#### 3.8.4. Colour Determination

Colour characteristics of bird cherry flour and the protein preparation were evaluated using a Konica Minolta CM-5 spectrophotometer (Konica Minolta, Tokyo, Japan) under standardized lighting conditions (D65). Measurements were expressed in the CIE Lab* colour system, where L* represents lightness on a scale from 0 (black) to 100 (white), a* indicates the red–green axis (negative values correspond to greenness, positive values to redness), and b* indicates the yellow–blue axis (negative values correspond to blueness, positive values to yellowness). The instrument was calibrated using a white reference plate before analysis. Each sample was measured in five replicates, and the mean values of L*, a*, and b* were calculated. From these coordinates, hue angle (h*) and chroma (C*) were computed using the following formulas [56]:

(4) $$Hue\;angle=Arctan\frac{b^{*}}{a^{*}}$$

(5) $$Chroma=\sqrt{{\left(a^{*}\right)}^{2}+}{\left(b^{*}\right)}^{2}$$

Chroma (C*) reflects the colour saturation or intensity, indicating whether the colour is vivid or more muted (C* = 0 corresponds to a neutral colour, higher C* values indicate stronger saturation). Hue angle (h*) provides information on the type of colour by combining the a* and b* coordinates, allowing for comparison of colour tones between different samples.

### 3.9. Statistical Analysis

Statistical analysis of all data was performed using one-way analysis of variance (ANOVA). The analysis of the chemical composition and functional properties was analyzed in triplicates, of the amino acid and fatty acid profiles in duplicate, and the colour measurements in five replicates. Duncan’s multiple range test was applied to identify significant differences among the samples at a probability level of 0.05. Statistical analysis and standard deviations were calculated using Statistica v. 13.3 software (Dell Software Inc., Round Rock, TX, USA).

All quantitative analyses, including ABTS radical scavenging activity, total polyphenols, amygdalin content, amino acid composition, and fatty acid profile, were conducted using appropriate reference standards. Calibration curves were prepared using authentic standards (e.g., Trolox for ABTS, gallic acid for total polyphenols, amygdalin standard for HPLC, certified amino acid and fatty acid standards), and all samples were analyzed in duplicate or triplicate to ensure accuracy, reproducibility, and comparability of the results.

## 4. Limitations of the Study

While this study provides a comprehensive analysis of the chemical composition, bioactive compounds, and functional properties of bird cherry (*Prunus padus* L.) flour and protein preparation, several limitations should be acknowledged. The protein isolation method, although effective, resulted in only moderate protein enrichment, and the purity of the isolated fraction was not verified with advanced techniques such as SDS-PAGE. Antioxidant activity was assessed solely by the ABTS assay, and the inclusion of complementary methods could offer a more complete evaluation of antioxidant potential and protein–polyphenol interactions. Moreover, the presence of erucic acid and residual amygdalin highlights the need for further toxicological assessment to ensure safe consumption. Functional properties were tested under controlled laboratory conditions, which may not fully represent their behavior in complex food matrices. Additionally, the lack of precise information on the origin and harvest conditions of the raw material may limit reproducibility. These considerations suggest that while the protein preparation shows promise for functional food applications, further optimization, detailed characterization, and safety studies are necessary to support its practical use.

## 5. Conclusions

Protein preparations obtained from bird cherry (*Prunus padus* L.) seeds are significantly enriched in protein, dietary fiber, and essential amino acids compared to the flour, while sugar and amygdalin contents are markedly reduced, thereby enhancing both nutritional value and safety. Phenolic compounds were largely retained, and antioxidant activity remained moderate, indicating that the extraction process effectively preserves bioactive components. Functional analyses demonstrated high protein solubility, water-binding capacity per protein unit, and improved emulsion stability, suggesting potential applications in various food formulations. The amino acid profile, particularly high levels of branched-chain amino acids and glutamic acid, supports the use of the preparation in high-protein and functional foods targeted at specific populations. The lipid fraction, dominated by long-chain monounsaturated fatty acids such as erucic acid, offers technological advantages but also necessitates careful evaluation of intake and long-term safety.

Overall, bird cherry protein preparations represent a promising plant-based ingredient for nutritionally enriched and functional food products, and further studies should focus on optimizing extraction methods, assessing protein purity, evaluating bioavailability, and ensuring the safety of fatty acids in long-term consumption.

## Figures and Tables

**Table 1:** Chemical composition of bird cherry flour and protein preparation.

	Flour	Protein Preparation
	g/100 g	
Dry matter	91.26 ± 0.02 ^b^	96.58 ± 0.19 ^a^
Total protein	15.44 ± 0.21 ^b^	39.72 ± 0.21 ^a^
Fat	11.76 ± 0.01 ^a^	8.94 ± 0.12 ^b^
Ash	4.78 ± 0.01 ^b^	8.53 ± 0.01 ^a^
Carbohydrates	59.28 ± 0.11 ^a^	39.39 ± 0.16 ^b^
Fibre	9.9 ± 0.01 ^b^	36.32 ± 0.01 ^a^
Total sugars	16.88 ± 0.01 ^a^	1.09 ±0.03 ^b^
Reducing sugars	12.69 ± 0.02 ^a^	0.82 ±0.02 ^b^

Values are means ± standard deviation. n = 3; ^a,b^ the same letters in the rows mean homogeneous groups (Duncan’s test *p* ≤ 0.05). Carbohydrates were calculated by difference and therefore include total sugars.

**Table 2:** Total phenolic content (TPC), antioxidant capacity (ABTS assay), and amygdalin content in bird cherry flour and protein preparation.

Analysed Material	TPC (mg GAE/g DM)	ABTS (µmol Trolox/g DM)	Amygdalin (mg/g)
Flour	15.38 ± 0.07 ^a^	26.36 ± 2.27 ^a^	1.72 ± 0.01 ^a^
Protein preparation	15.31 ± 0.02 ^a^	29.26 ± 2.48 ^a^	0.15 ± 0.01 ^b^

Values are means ± standard deviation. n = 3; ^a,b^ the same letters in the columns mean homogeneous groups (Duncan’s test *p* ≤ 0.05).

**Table 3:** Amino acid profile of bird cherry flour and protein preparation.

Amino Acid	Flour	Protein Preparation
	mg/g	
IAA *		
Leucine	9.80 ± 0.06 ^b^	35.12 ± 0.53 ^a^
Isoleucine	5.97 ± 0.04 ^b^	19.01 ± 0.36 ^a^
Methionine	2.88 ± 0.05 ^a^	2.57 ± 0.10 ^a^
Cysteine	0.33 ± 0.05 ^a^	0.18 ± 0.17 ^a^
Phenylalanine	5.32 ± 0.01 ^b^	22.10 ± 0.42 ^a^
Threonine	4.55 ± 0.28 ^b^	13.89 ± 0.38 ^a^
Lysine	10.07 ± 0.06 ^b^	14.84 ± 0.24 ^a^
Tyrosine	2.54 ± 0.01 ^b^	12.07 ± 0.26 ^a^
Valine	5.86 ± 0.20 ^b^	22.40 ± 0.50 ^a^
DAA **		
Aspartic acid	21.14 ± 0.89 ^b^	36.43 ± 0.82 ^a^
Glutamic acid	24.96 ± 0.66 ^b^	74.41 ± 1.25 ^a^
Serine	5.92 ± 0.35 ^b^	14.63 ± 0.48 ^a^
Glycine	9.11 ± 0.23 ^b^	22.55 ± 0.42 ^a^
Alanine	4.50 ± 0.08 ^b^	16.19 ± 0.45 ^a^
Histidine	3.51 ± 0.44 ^a^	2.71 ± 0.07 ^b^
Arginine	5.88 ± 0.91 ^b^	17.90 ± 0.72 ^a^
Proline	5.64 ± 0.57 ^a^	18.41 ± 0.15 ^a^
Total amino acids	122.05 ± 0.26 ^b^	345.40 ± 6.97 ^a^

Values are means ± standard deviation. n = 2; ^a,b^ the same letters in verse mean homogenous groups (Duncan’s test *p* ≤ 0.05); IAA *—indispensable amino acids; DAA **—dispensable amino acids.

**Table 4:** Fatty acid composition of bird cherry flour and protein preparation.

Fatty Acid	Flour	Protein Preparation
	% of Total Fatty Acid Profile	
Palmitic acid (C16:0)	1.42 ± 0.01 ^b^	2.24 ± 0.01 ^a^
Stearic acid (C18:0)	0.22 ± 0.01 ^b^	2.72 ± 0.01 ^a^
Oleic acid (C18:1)	5.35 ± 0.01 ^b^	6.65± 0.01 ^a^
Linoleic acid (C18:2)	0.79 ± 0.01 ^b^	1.66 ± 0.01 ^a^
α-linolenic acid (C18:3n3)	0.91 ± 0.01 ^b^	2.17 ± 0.01 ^a^
Arachidic acid (C20:0)	0.15 ± 0.01 ^a^	0.16 ± 0.01 ^a^
Eicosenoic acid (C20:1)	15.51 ± 0.01 ^b^	16.61 ± 0.01 ^a^
Eicosadienoic acid (C20:2)	0.06 ± 0.01 ^a^	0.07 ± 0.00 ^a^
Eicosatrienoic acid (C20:3n3)	0.06 ± 0.01 ^a^	0.06 ± 0.01 ^a^
Arachidonic acid (C20:4)	0.07 ± 0.00 ^b^	0.10 ± 0.00 ^a^
Erucic acid (C22:1)	74.55 ± 0.01 ^a^	68.16 ± 0.01 ^b^
Docosadienoic acid (C22:2)	0.10 ± 0.00 ^a^	0.12 ± 0.01 ^a^
Lignoceric acid (C24:0)	0.22 ± 0.01 ^a^	0.23 ± 0.01 ^a^
Nervonic acid (C24:1)	1.54 ± 0.01 ^a^	1.51 ± 0.01 ^a^
∑ SFA	2.01	5.34
∑ MUFA	96.94	92.92
∑ PUFA	1.98	4.16

Values are means ± standard deviation. n = 2; ^a,b^ the same letters in the rows mean homogeneous groups (Duncan’s test *p* ≤ 0.05). MUFA monounsaturated fatty acids, PUFA polyunsaturated fatty acids, SFA saturated fatty acids.

**Table 5:** Chosen functional properties of bird cherry flour and protein preparation.

Functional Property	Flour	Protein Preparation
PSI (%)	28.4 ± 1.20 ^b^	76.8 ± 2.10 ^a^
WAC (g water/g preparation)	2.72 ± 0.11 ^a^	2.15 ± 0.07 ^b^
WAC (g water/g protein)	0.42 ± 0.11 ^b^	0.85 ± 0.07 ^a^
OAC (ml oil/g preparation)	5.42 ± 0.07 ^a^	1.67 ± 0.05 ^b^
OAC (ml oil/g protein)	0.84 ± 0.07 ^a^	0.66 ± 0.05 ^a^

Values are means ± standard deviation. n = 3; ^a,b^ the same letters in the verse mean homogeneous groups (Duncan’s test *p* ≤ 0.05). PSI—protein solubility index; WAC—water-absorption capacity; OAC—oil-absorption capacity.

**Table 6:** Emulsifying properties of bird cherry flour and protein preparation.

Emulsifying Properties	EA	ES
	%	
Flour	82.22 ± 0.12 ^a^	35.56 ± 0.07 ^b^
Protein preparation	47.53 ± 0.22 ^b^	63.22 ± 0.08 ^a^

Values are means ± standard deviation. n = 3; ^a,b^ the same letters in the verse mean homogeneous groups (Duncan’s test *p* ≤ 0.05). EA—emulsifying activity; ES—emulsifying stability.

## Data Availability

Data is contained within the article.
